# Nematicidal potential of *Microbacterium maritypicum* Sneb159 against *Heterodera glycines* and the complete genome sequence analysis

**DOI:** 10.3389/fpls.2025.1485160

**Published:** 2025-02-07

**Authors:** Jing Zhao, Zhifu Xing, Aatika Sikandar, Yuxi Duan

**Affiliations:** ^1^ School of Breeding and Multiplication (Sanya Institute of Breeding and Multiplication), Hainan University, Sanya, China; ^2^ School of Tropical Agriculture and Forestry, Hainan University, Danzhou, China; ^3^ College of Plant Protection, Shenyang Agricultural University, Shenyang, China

**Keywords:** *Heterodera glycines*, *Microbacterium maritypicum*, nematicidal activity, volatile organic compound, complete genome

## Abstract

**Introduction:**

*Heterodera glycines* is one of the most important pathogens of soybean production worldwide. Biological control provides a strategy for sustainable and environmentally friendly nematode management.

**Methods:**

In this study, solid-phase microextraction gas chromatography-mass spectrometry (SPME-GC-MS) was used to reveal the volatile nematicidal compounds of *Microbacterium maritypicum* Sneb159 and the mode of action was further elucidated via whole genome sequencing.

**Results:**

The present study demonstrated that *M. maritypicum* Sneb159 fermentation broth showed strong nematicidal activities against *H. glycines*. The filtrate rather than bacterial cells played a role in the nematicidal character, and reduced the invasion number as well as suppressed the development of juveniles in soybean. The analysis of chemotaxis showed that *M. maritypicum* Sneb159 has a repellent effect on *H. glycines* in pot experiments. The volatiles produced by *M. maritypicum* Sneb159 are toxic to *H. glycines* for both juveniles and eggs. The seven compounds were analyzed using SPME-GC-MS. In the bioassays, dimethyl disulfide and dimethyl trisulfide showed both direct contact and fumigated effect on juveniles and eggs. The complete genome sequence of the bacterium *M. maritypicum* Sneb159 was completed using the PacBio sequencing platform. The genome comprised 3895529 bp and a 68.63% G + C content. Three secondary metabolites gene clusters were predicted by the antiSMASH system.

**Discussion:**

The findings reveal multifunction of *M. maritypicum* Sneb159 towards *H. glycines*, and has the potential to be developed as a safe nematicidal agent.

## Introduction

The soybean (Glycine max L.) is an essential crop, providing a primary source of protein and oil. As the fourth largest producer of soybeans, China produced 1.75 million tons of crops in 2020 (Peng et al., 2021). Plant-parasitic nematodes (PPNs) are widespread biotic stress of plants, causing significant quantitative and qualitative economic losses in a wide range of crops. The estimated global annual losses attributed to PPNs have been estimated to range from 80 to 157 billion dollars (Ansari and Saleem, 2023). *Heterodera glycines* is one of the most destructive PPNs of soybean crops, drastically impeding crop yield. In China, the distribution of *H. glycines* covers 22 provinces, including Heilongjiang, Zhejiang, and Guangxi (Zheng et al., 2009; Peng et al., 2021). The annual yield loss attributable to SCN in China is estimated to exceed $120 million (Ou et al., 2008). *H. glycines* destroys the physical structure of soybean roots, and absorbs nutrients from them. It is unfeasible to eradicate, once soybean fields have been heavily infested. Currently, using the rotation of non-host plants, resistant soybean varieties, and appropriate agricultural practices could mitigate the disease (Arjoune et al., 2022). Chemical control using nematicides is available for SCN management. However, nematicides are costly to use, and there’s little evidence that the yield increase is significant enough to support their use (Niblack et al., 2006). Besides, high doses are usually required, and the long-term application causes serious environmental pollution, and poses a severe threat to the safety of humans and animals. Biocontrol is a promising approach to managing SCN, typically involving multiple mechanisms such as the production of toxins or antibiotics, the secretion of degrading enzymes, the induction of systemic resistance and the promotion of soybean growth (Tian et al., 2000; Zhou et al., 2024). What’s more, some biocontrol agents have been reported to reconstruct the composition of rhizosphere microorganisms, which has profound implications for maintaining soil health (Jin et al., 2021). Currently, biocontrol is one of the most important methods of soybean disease control, and it’s expected to have an extensive application prospect in the future (Yu et al., 2022).

Bacterial antagonists are the main resource and have been investigated for several years for their utilization as biocontrol agents against plant-parasitic nematodes (Forghani and Hajihassani, 2020). Hu et al. (2022) studied the control of *Meloidogyne incognita* with *Bacillus velezensis* strain YS-AT-DS1 in tomatoes and showed a mortality rate of 71.62% as well as a significant reduction in infection, galls and egg masses. In recent years, researchers have focused on exploring natural products that can replace the chemical nematicides. *Streptomyces* sp. AN091965 produced spectinabilin, which showed significant nematicidal activity against *Bursaphelenchus xylophilus*, effectively suppressing the development of pine wilt disease (Liu et al., 2019). Volatile organic compounds (VOCs) which are produced by plants or microorganisms, are considered to be potential substances for fumigants (Deng et al., 2022). Bacteria release multiple volatiles, including alcohols, alkenes, acids, esters, ketones, and terpenes (Peñuelas et al., 2014). *B. cereus* Bc-cm103 showed nematicidal activity to *M. incognita*, and the nematicidal VOCs were dimethyl disulfide and S-methyl ester butanethioic acid (Yin et al., 2021). Dimethyl disulfide and acetaldehyde were isolated from the deep-sea bacterium *Virgibacillus dokdonensis* have contact and attraction activity to *M. incognita* (Huang et al., 2020). Recent studies have further revealed the effect of some common bacteria such as *Bacillus* sp., *Pseudomonas* sp., *Klebsiella* sp. and *Streptomyces* sp. in the control of root-knot nematodes, the exploration of more novel biocontrol agents for cyst nematodes and the investigation of the mechanism of action is urgent in the context of such rich microbial resources.

*Microbacterium maritypicum* was first isolated from sea and marine mud (Takeuchi and Hatano, 1998). The potential of the gram-positive bacterium to tolerate abiotic stress has been discussed, including UV irradiation resistance and salt tolerance (Williams et al., 2007). Besides, *M. maritypicum* ABR5 was considered a biosurfactant-producer for the recovery of oil from oily sludge in the petroleum industry (Akbari et al., 2020). However, it is a relatively novel microbial resource for nematode control. Our previous research showed that *M. maritypicum* Sneb159 induced resistance in soybean by activating the response of the SA and JA signaling pathways and effectively controlled *H. glycines* (Zhao et al., 2019). However, there were no reports about the direct action of *M. maritypicum* against *H. glycines*, such as the nematocidal activity of metabolites and VOCs and the effect on chemotaxis.

This study investigated the toxicity of bacterial cells and fermentation filtrate and the negative effect on the infection and development of *H. glycines*. The nematicidal action of VOCs on juveniles and eggs was confirmed, and analyzed the composition of VOCs. Moreover, we presented the annotation of *M. maritypicum* Sneb159 genome draft, and analyzed the secondary metabolite biosynthetic gene clusters. These results will reveal a more comprehensive mechanism of *M. maritypicum* Sneb159 on cyst nematodes, and provide a theoretical basis for future application.

## Materials and methods

### Preparation of eggs and second-stage juveniles

*H. glycines* cysts were obtained from infected soybean roots and rhizosphere soil by massaging in water and sieving the solution through a set of 850 μm and 250 μm pore-sized nested sieves. Cysts retained on the 250-μm sieve were purified using the sucrose flotation method (Krusberg et al., 1994). Subsequently, the cysts were crushed and passed through with 45-μm and 25-μm pore-size sieves. Eggs recovered from the 25-μm sieve were surface-sterilized with 0.5% (w/v) NaClO and rinsed several times with sterile distilled water (SDW). Sterilized eggs were incubated in a Baermann funnel at 26°C, and the second-stage juveniles (J2s) were collected daily.

### Preparation of culture filtrates of *M. maritypicum* Sneb159

Strain Sneb159 was isolated from the crop rhizosphere soil, identified as *M. maritypicum* and stored at -80°C (Zhao et al., 2019). Activated Sneb159 was cultured in 100 mL lysogeny broth (LB) at 200 rpm at 28°C for 72 h. Bacterial cells were diluted to 10^8^ CFU/mL (OD600 = 0.8) with SDW. Culture filtrates were achieved via centrifugation at 12,000 rpm for 10 min and passed through a 0.22-μm filter to remove additional bacterial cells. Bacterial cells were diluted with SDW to a concentration of 10^8^ CFU/mL.

### Nematicidal activity test against *H. glycines*

To evaluate the nematicidal activity of Sneb159 on the J2 of *H. glycines*, 1 mL of fermentation or LB broth was added to a 2 mL centrifuge tube containing 50 J2s. Besides, the supernatant or bacterial cells of 10^8^ CFU/mL were diluted 10-fold and 100-fold with SDW, respectively. The number of dead nematodes was recorded after 24, 48, or 72 h under a stereomicroscope (Nikon SMZ800, Japan). The experiment was conducted three times with three replications. Nematodes were treated with culture filtrates and bacterial cells diluted 10-fold and 100-fold, and mortality was determined after 48 h. The experiment was conducted twice with three replications. Nematodes were considered dead if they were rigid and did not respond to a small needle probe. The mortality rate was calculated using the following equation:


[(live J2s before exposure–live J2s after incubation) / live J2s before exposure]×100

### Pot experiments

Soybean seeds (Liaodou15) were disinfected with 1% NaClO for 5 min and washed at least three times with distilled water. An individual soybean seed was cultivated in a 12 cm diameter plastic pot filled with 300 g of sterile sand. At the third true leaf stage, 10 mL of Sneb159 bacterial cells (culture filtrates diluted 0, 10, or 100-fold) were injected around the root. Subsequently, 2000 J2s were inoculated after 48 h. The number of juveniles inside the roots was measured at 5 days post infection (dpi) and 15 dpi by using the acid fuchsin staining method. The experiment was performed twice, and each treatment consisted of five replicates.

### Chemotaxis of *H. glycines* in pot experiments

The chemotaxis in pot experiments was designed as described by Wang et al. (2019). A total of 250 mL of sterilized sand was filled in each of the plastic cups (9 × 15 cm). Plastic cups containing soybean seeds were grown in a greenhouse at 26°C for a 16-h photoperiod. A 5 mL liquid culture of Sneb159 or sterile distilled water was added to the opposite plastic cups when the seedlings had developed the third true leaf, and both sides added with sterile distilled water were considered blank controls. Two plastic cups were connected with a polyvinyl chloride tube (1.5 cm diameter, 7 cm length). The tube was filled with sterilized sand, and a hole (1 cm in diameter) was drilled in the middle. The J2s (about 1000) were injected through the hole and wrapped in tin foil for protection from light. The roots were collected, stained with acid fuchsin at 10 dpi, and observed using a stereomicroscope. The experiment was conducted twice with eight replications.

### Activity of *M. maritypicum* Sneb159 VOCs against *H. glycines in vitro*

The nematicidal activities of Sneb159 and Sneb517 VOCs were assayed using a three-cell Petri dish method described by Zhai et al. (2018) with minor modifications. In brief, 2.5 mL of culture filtrates were added to one compartment, and a layer of 2.5 mL of 2% water agar was added to another compartment with 50 μL of approximately 100 *H. glycines* J2 on the surface. Control plates contained LB instead of the culture filtrates. Activated charcoal was added to the third compartment to absorb the VOCs generated by Sneb159 and confirm that VOCs paly a main role in nematicidal activity. The plates were immediately sealed and cultured at 26°C. The experiment was conducted three times with three replications. The mortality was measured under a stereomicroscope (Nikon SMZ800, Japan) after 72 h.

Three linked wells of a 96-well plate were used to determine the effects of the Sneb159 VOCs on egg hatching. A drop of 300 μL of Sneb159 culture filtrate was added to the middle well. Subsequently, approximately 50 eggs (100 μL) were added to the other two wells. SDW and LB were used as controls. The hatchability rate was investigated at 9 d after incubation. The experiment was conducted three times with four replications.

### Identification of volatile compounds

Sneb159 was cultured in 100 mL LB at 200 rpm at 28°C for 72 h. A total of 9 mL fermentation broth was added to a 20-mL headspace vial. The VOCs were extracted from the headspace vials of inoculated and uninoculated broths using a 65-mm PDMS/DVB SPME fiber (Supelco Inc., Bellefonte, PA, USA). Specifically, the SPME fiber pierced the PTFE septum of the headspace vial and was exposed above the liquid for 1 h while the vial was immersed in a thermostatic water bath at 60°C. Immediately after VOC extraction, the fiber was inserted into the gas chromatograph front inlet and desorbed at 250°C for 5 min. The experiment was conducted using an Agilent 6890N gas chromatograph (Agilent Technologies, Santa Clara, CA, USA) that was connected with an Agilent 5973N mass spectrometer and a DB-5 capillary column (30 m × 0.25 mm × 0.25 μm).

The flow rate of the carrier gas (helium) was 1 mL/min in splitless mode. The oven parameters were set according to (Zhai et al., 2018) and were as follows: 40°C for 2 min, raised to 180°C at 4°C/min rate, and then raised to 250°C at 5°C/min rate, and held for 6 min. The transfer line and ion source temperatures were 150°C and 230°C, respectively. The MS detector ranged between 35 and 520 *m/z*. The identification of compounds was achieved by using the National Institute of Standards and Technology (NIST98) reference library and the comparison of the retention times. The VOCs from the LB medium were considered the control group. The unique substances produced by strain Sneb159 were identified after eliminating the substances also detected in the LB medium. Each sample was measured twice.

### Contact inhibition activity of commercial VOCs

For nematicidal tests, commercial dimethyl disulfide, dimethyl trisulfide, and cyclohexyl(dimethoxy)methylsilane were used. The chemical solution was diluted to 50-800 mg/L with 0.03% Tween-80 (v/v). The 0.5 mL centrifuge tubes containing 10 μL *H. glycines* J2s (approximately 50 J2s) were filled with different concentrations of test solutions (300 μL). J2s soaked in 0.03% Tween-80 (v/v) solution were used as the control group. The mortality rate was measured after incubation for 24 and 48 h at 26°C. The experiment was conducted three times with three replications.

In order to determine the effect of commercial VOCs on hatchability, 50 eggs suspended in 10 μL SDW were placed in 0.5-mL centrifuge tubes containing 300 μL test solutions. The concentration of dimethyl disulfide and dimethyl trisulfide ranged from 100 to 800 mg/L. The 0.03% Tween-80 (v/v) solution was used as a control. Hatchability was calculated after 9 d at 26°C. Each treatment was conducted in triplicate with three replications.

### Fumigant activity of commercial VOCs

The fumigation experiments were performed using a 96-well plate. 300 μL of commercial VOC was added to one cell of a 96-well plate, and a 100 μL suspension of nematodes or eggs (approximately 50) was dropped into the two adjacent wells. The final concentration of commercial VOCs was adjusted to 200, 400, or 800 mg/L using 0.03% Tween-80 (v/v). The three wells were sealed with parafilm, and 0.03% Tween-80 (v/v) was used as a control. The number of dead nematodes was counted after 72 h at 26°C. The percentage of hatched eggs was recorded after 9 d at 26°C. The experiment was conducted three times with three replications.

### Genome sequencing, assembly, and annotation

For sequencing, the total DNA of M. maritypicum Sneb159 was extracted using E.Z.N.A^®^ Bacteria DNA kit (Omega). Pair-end libraries were prepared following the manufacturer instructions with the TruSeq™ Nano DNA Sample Prep Kit (Illumina, USA), and sequenced on the Pacific Biosciences Sequel II technology (PacBio) and the Illumina NovaSeq 6000 platform (150 bp×2, Shanghai Biozeron Biotechnology Co., Ltd., Shanghai, China). The raw paired-end reads were trimmed and quality controlled by Trimmomatic with Parameters (SLIDINGWINDOW: 4: 15 MINLEN: 75) (v0.36; http://www.usadellab.org/cms/uploads/supplementary/Trimmomatic). The collected clean data was used for further analysis. Raw PacBio reads were converted to fastq format with Samtools fastq (http://www.htslib.org/doc/samtools.html). The Illumina data was used to evaluate the complexity of the genome and correct the PacBio long reads. First, we used unicycler v0.4.8 (https://github.com/rrwick/Unicycler) to perform genome assembly with default parameters and received the optimal results of the assembly. GC depth and genome size information were calculated by custom Perl scripts (Biozeron Biotechnology Co., Ltd), which can help us judge whether the DNA sample contained contaminated or not. Finally, the strain genome was circularized with Circlator v1.5.5 (http://sanger-pathogens.github.io/circlator/).

For the prokaryotic organism, we used the ab initio prediction method to get gene models for M. maritypicum Sneb159. Gene models were identified using GeneMark v4.17 (http://topaz.gatech.edu/GeneMark/). Then all gene models were blastp against non-redundant (NR in NCBI) databases, SwissProt (http://uniprot.org), KEGG (http://www.genome.jp/kegg/), and COG (http://www.ncbi.nlm.nih.gov/COG) to do functional annotation by the blastp module. In addition, tRNA was identified using the tRNAscan-SE (v2.04; http://lowelab.ucsc.edu/tRNAscan-SE), and rRNA was determined using the RNAmmer (v1.2; http://www.cbs.dtu.dk/services/RNAmmer/). Homologs to known virulence factors, transport proteins, antibiotic resistance genes, and metal resistance genes were identified using the following databases: the Virulence Factor Database (VFDB: https://www.mgc.ac.cn/VFs/main.htm), the Transporter Classification Database (TCDB: https://tcdb.org/), the Comprehensive Antibiotic Resistance Database (CARD: https://card.mcmaster.ca/), and the antibacterial biocide and metal resistance genes database (BacMet version 2.0: http://bacmet.biomedicine.gu.se/). The second metabolite biosynthetic gene cluster of M. maritypicum Sneb159 was predicted using the antiSMASH 7.0 platform (Blin et al., 2023).

### Statistical analysis

Nematicidal activity of Sneb159 fermentation broth and chemotaxis response in the pot experiment were tested with an independent sample *t*-test (*p*<0.05 or *p*<0.001). Nematicidal activity of filtrate, pot experiment, Sneb159 VOCs on juveniles and eggs was analyzed using one-way analysis of variance with Tukey’s *post hoc* test (*p*<0.05). Nematicidal activity data for commercial VOCs were corrected using the Schneider-Orelli formula (Ntalli et al., 2011), and LC_50_ values were calculated using PROBIT analysis (Cheng et al., 2017). All data are shown as the mean ± standard error. All statistical tests were performed using IBM SPSS Statistics 23 software (IBM Corp., Armonk, NY, USA).

## Results

### Nematicidal activity of *M. maritypicum* Sneb159 on nematodes

**Figure 1:**
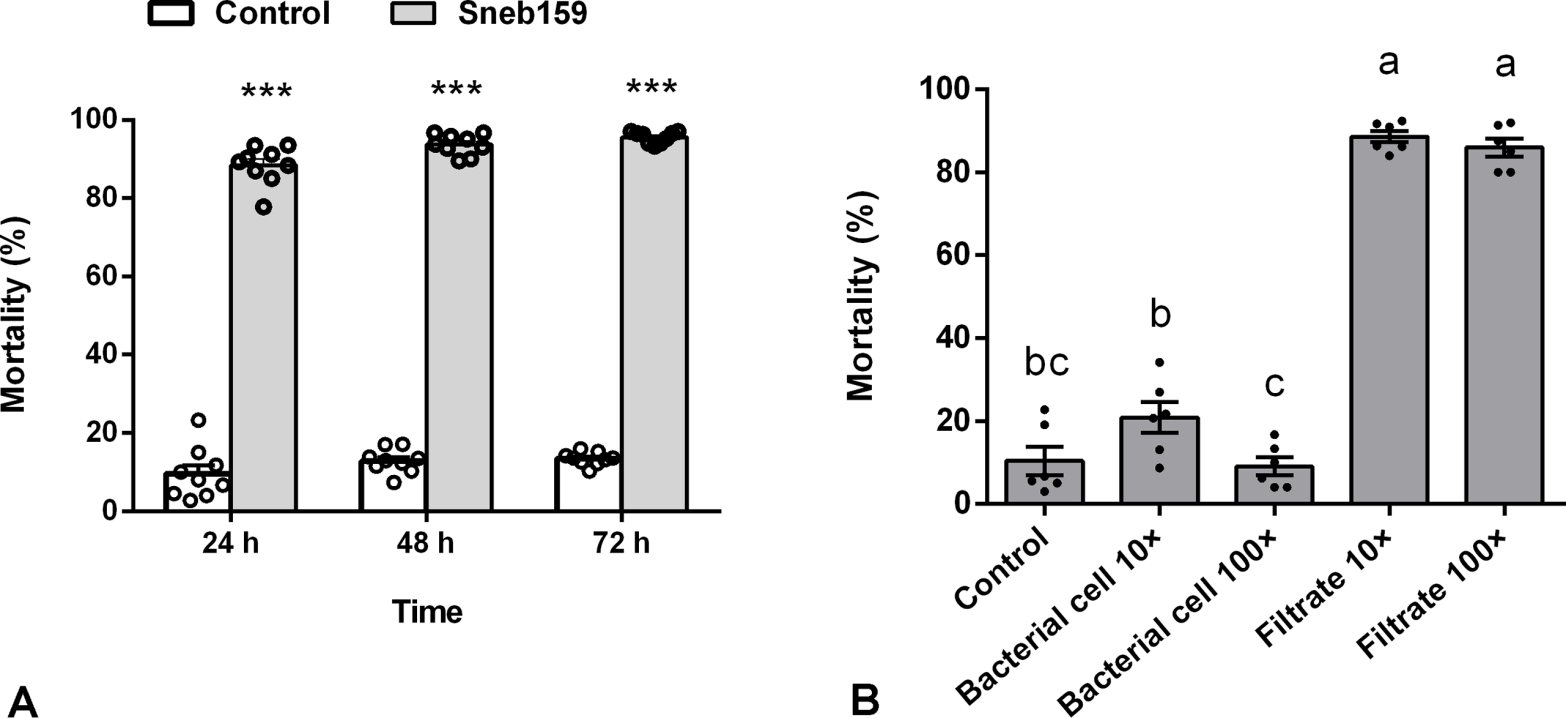
*In vitro* nematicidal effect of *Microbacterium maritypicum* Sneb159. **(A)** The mortality of *Heterodera glycines* after treated with fermentation broth of *M. maritypicum* Sneb159. **(B)** The mortality of *H*. *glycines* in the treatment of bacterial cells or filtrates. The asterisk represents a significant difference using *t*-test, ***means *p*<0.001; the different letters on the bars mean significant different according to Tukey’s *post hoc* test (*p*<0.05).

The fermentation of Sneb159, cultivated in LB medium, showed strong nematicidal activity against J2 of *H. glycines* with mortalities of 88.43, 93.7, and 95.53% at 24, 48, and 72 h, respectively. The mortality rate for the LB treatment was 9.57, 12.88, and 13.45% at 24, 48, and 72 h, respectively, which were all significantly lower than those of the Sneb159 treatment according to the *t*-test (*P*<0.001) (**Figure 1A**). Moreover, the nematicidal activity of fermentation filtrate and bacterial cells against nematodes was determined after separation. The results revealed that the mortality rates for fermentation filtrate diluted 10 and 100 folds were 88.57, and 85.95%, which were significantly higher than the control using Tukey’s *post hoc* test (*p*<0.05) (**Figure 1B**). However, no notable nematicidal properties were observed in the bacterial cell treatment, both bacterial cells diluted 10 and 100 folds with no significant difference compared to the control (*P*<0.05).

### *In vivo* effect of *M. maritypicum* Sneb159 in pot experiments

**Figure 2:**
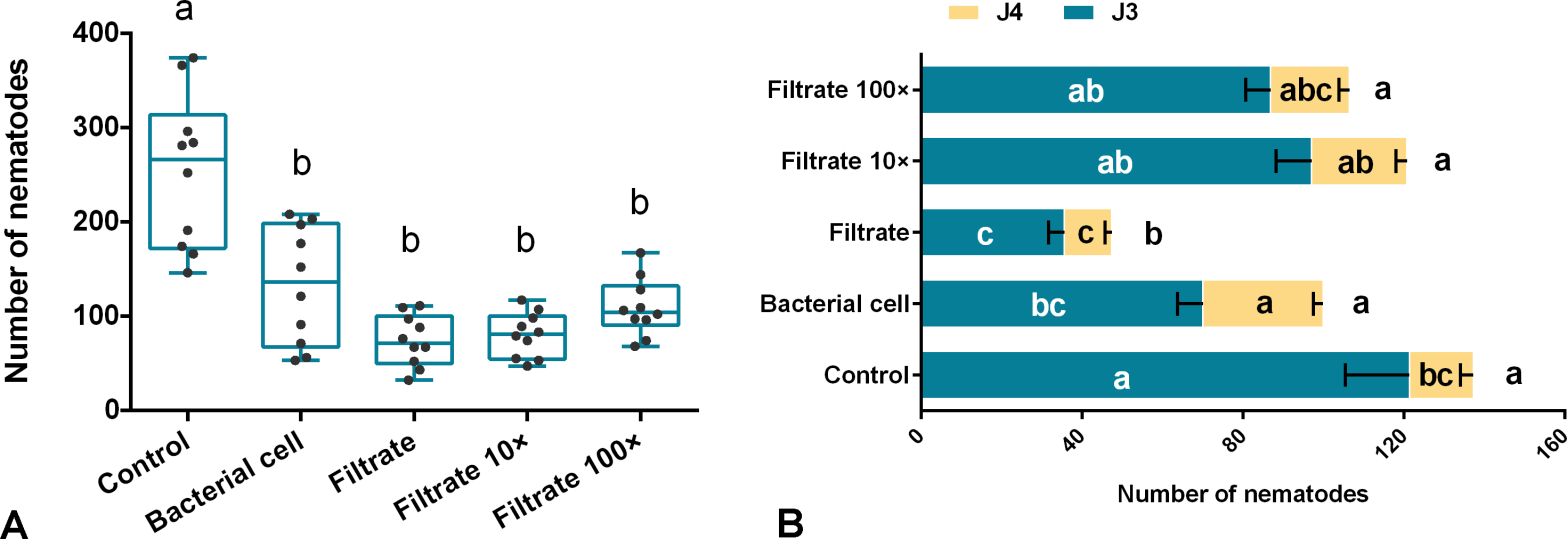
*In vivo* biological effect of *Microbacterium maritypicum* Sneb159 on the invasion and development of *Heterodera glycines*. **(A)** The number of infected juveniles at 5 dpi. **(B)** Effect of *M. maritypicum* Sneb159 on nematode development. The different letters on the bars mean significant different according to Tukey’s *post hoc* test (*p*<0.05).

The effect of Sneb159 on the invasion and development of *H. glycines* juveniles was evaluated by pot experiments. At 5 dpi, pre-inoculation with bacterial cells or filtrate led to a significant reduction in the number of infected J2s (*P*<0.05). Compared with the control, the reduction in the filtrate, filtrate dilution 10-fold, and filtrate dilution 100-fold was 70.67%, 68.3% and 56.88% respectively. The inhibitory effect decreased as the dilution ratio increased (**Figure 2A**). However, no differences were observed among bacterial cells, filtrate, 10-fold and 100-fold filtrate.

At 15 dpi, the number of different stages of juveniles was under investigation. Most juveniles have already developed to the third and fourth stages of juveniles (J3 and J4) (**Figure 2B**). Both J3 and the total number of juveniles in filter-treated roots were remarkably decreased compared to the control (*p*<0.05), with reduction rates of 70.60% and 65.43% accordingly. However, no statistical significance was observed when the filtrate was diluted 10 and 100-fold for the number of J3, J4, and total. After treatment with bacterial cells, the number of J3 was decreased by 42.27%, the J4 number increased by 46.65% compared to the control, and there was no significant difference in the total number of juveniles between them.

### Analyses of *H. glycines* behavior in pot experiments

**Figure 3:**
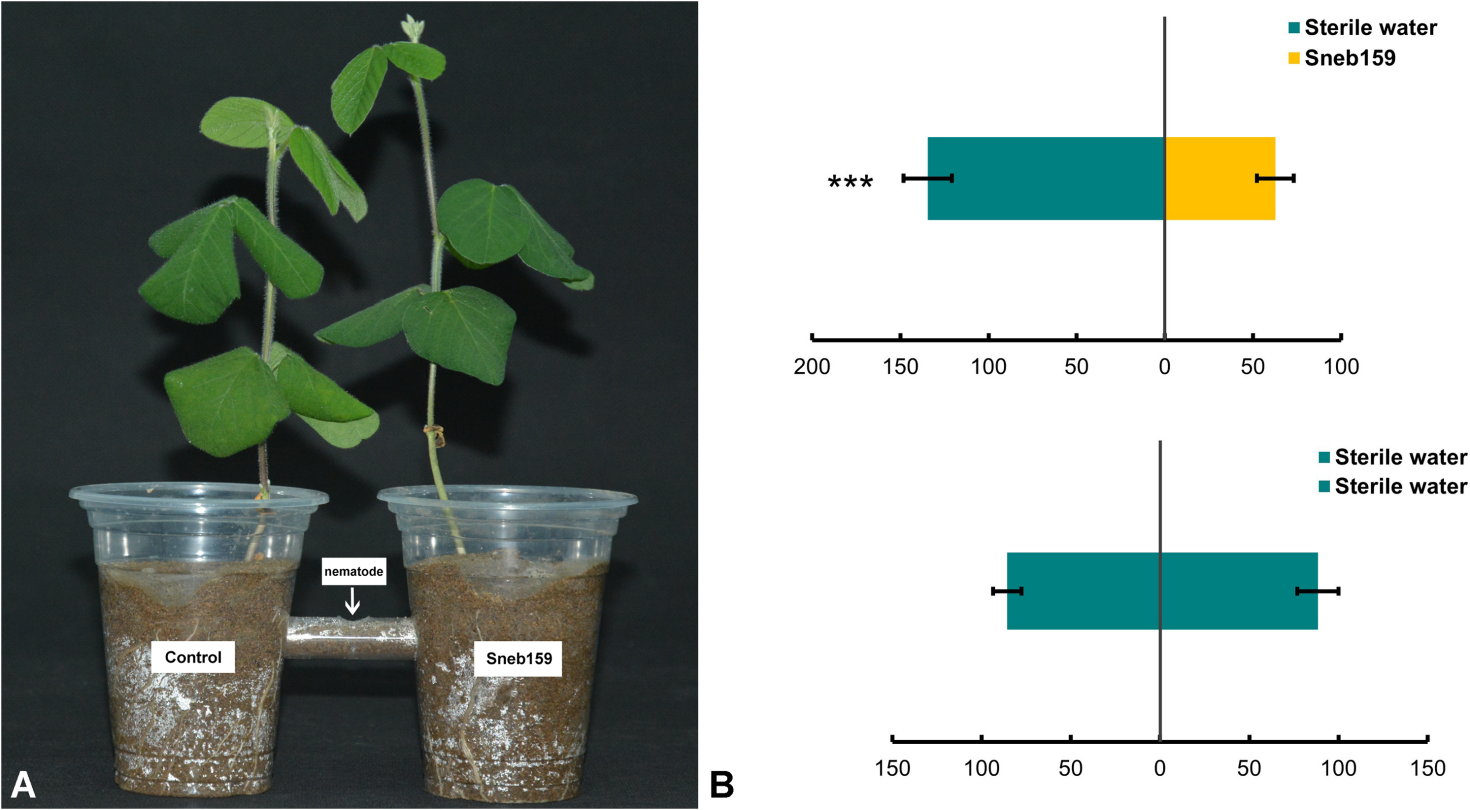
Chemotaxis test of *Heterodera glycines* under the treatment of *Microbacterium maritypicum* Sneb159. **(A)** The diagram of chemotaxis test. **(B)** *H*. *glycines* response to *M. maritypicum* Sneb159 in the pot assay. The asterisk represents a significant difference using *t*-test, *** means *p*<0.001.

The effect of Sneb159 on nematode chemotaxis in soil was determined (**Figure 3A**). J2s were repelled by the Sneb159 fermentation broth, with more juveniles moving to the other side of the sterile water treatment. The greatest decrease was 53.41% compared to the control (*P* < 0.001) (**Figure 3B**). For control, there was no significant difference in the number of J2s between the two sides. No significant difference in total nematode numbers between the treatment group and the control group (*P* < 0.05) means that repelling rather than killing caused the reduction in nematodes on the Sneb159 side.

### Effect of *M. maritypicum* Sneb159 volatiles on *H. glycines*

**Figure 4:**
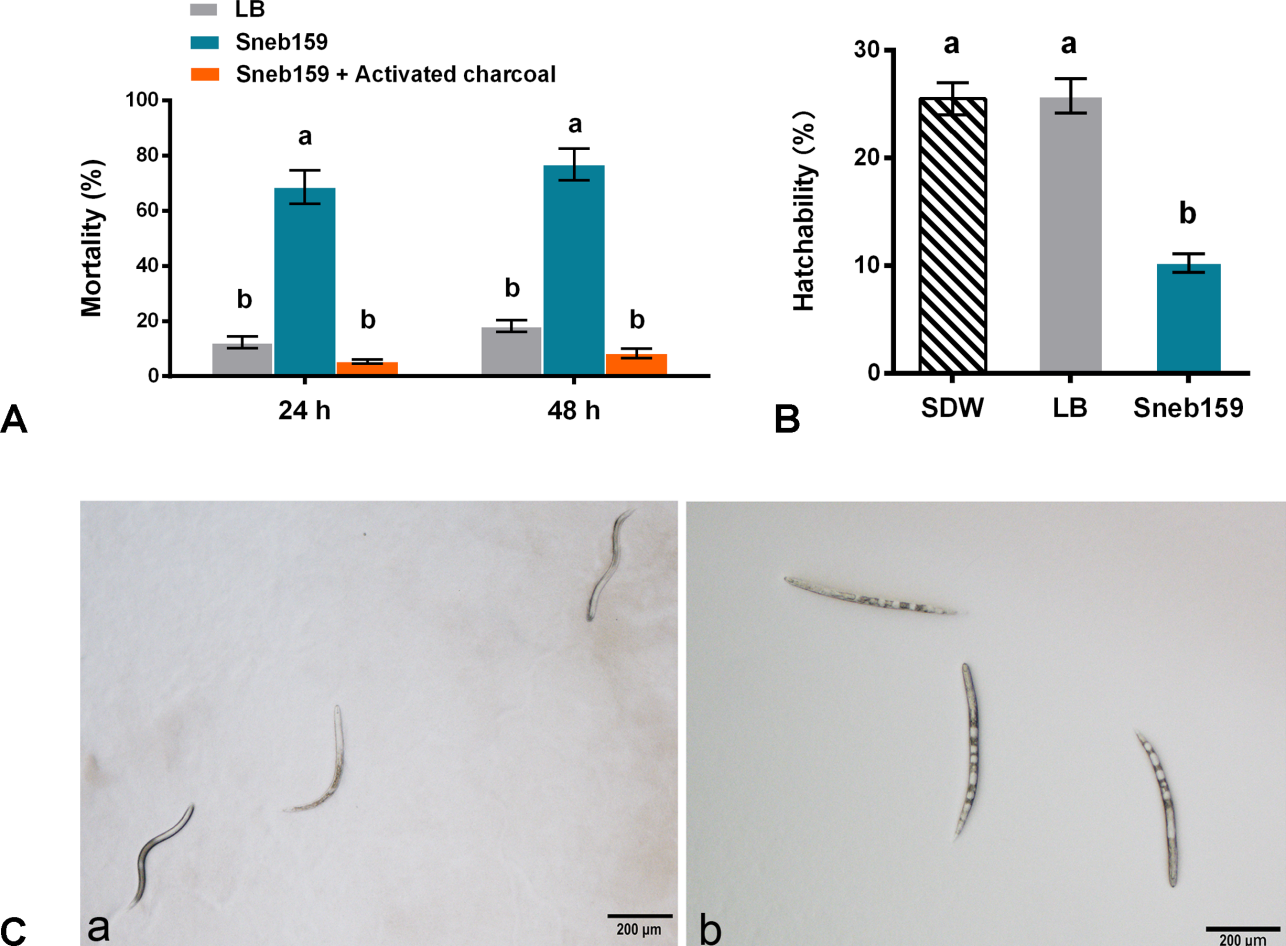
Fumigation effect of *Microbacterium maritypicum* Sneb159 volatiles on *Heterodera glycines in vitro*. **(A)** Mortality of *H*. *glycines* juveniles after exposure to volatiles of Sneb159. **(B)** Hatchability of *H. glycines* eggs after exposure to volatiles of Sneb159 after 9 days. **(C)** Morphology of *H. glycines* after LB control (a) and Sneb159 treatment (b). Different letters indicate significant difference according to Tukey’s *post hoc* test (*p*<0.05), and 24 h and 48 h treatments were analyzed separately.

To evaluate the influence of VOCs from *M. maritypicum* Sneb159 fermentation broth on *H. glycines* J2s, three-cell Petri dishes were used. After 24 h of incubation, the mortality in the Sneb159 VOCs was 68.61%, which was significantly higher than that in the LB control (12.35%) (**Figure 4A**). The mortality rate reached 76.84% in Sneb159 treatment after 48 h. Activated charcoal was added to absorb the volatiles, and the mortality was 5.33% and 8.28% after 24 and 48 h, respectively. The juveniles were coiled and mobile in the LB control at 48 h. While most of the juveniles in the Sneb159 treatment were stiff and immobile, their organs were destroyed and dissolved, and several severe vacuolations appeared in their bodies (**Figure 4C**). After 9 d, the egg hatchability of the Sneb159 volatiles treatment was 10.25%, which was greatly reduced compared to the LB control (25.81%) and the SDW control (25.54%) (*p*<0.05) (**Figure 4B**).

### Analysis of *M. maritypicum* Sneb159 volatiles via GC/MS

**Figure 5:**
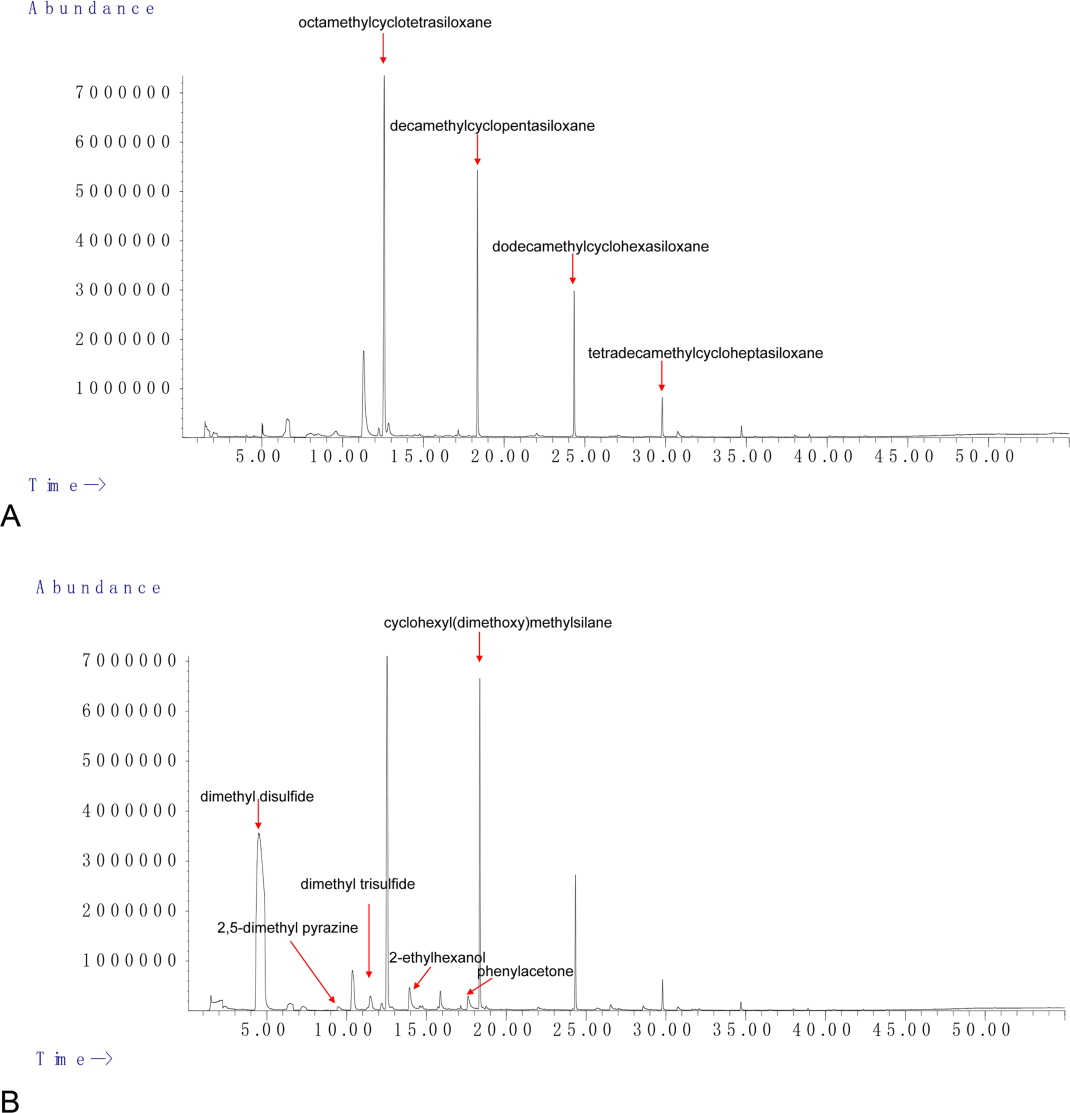
GC/MS chromatogram showing identified volatiles from LB control **(A)** and *Microbacterium maritypicum* Sneb159 **(B)**.

The specific components of the VOCs collected via headspace-solid phase microextraction were identified by GC/MS analysis (**Figure 5**). Seven compounds (Qual > 80) were identified in Sneb159 after the removal of the common substance in LB, including dimethyl disulfide, 2,5-dimethyl pyrazine, dimethyl trisulfide, 2-ethylhexanol, 3-ethyl-2,5-dimethylpyrazine, phenylacetone, and cyclohexyl(dimethoxy)methylsilane (**Table 1**). The retention of Sneb159 spanned from 4.483 to 30.755 minutes. Dimethyl disulfide was the most abundant compound in Sneb159, with a 50.25% relative area, followed by cyclohexyl(dimethoxy)methylsilane (10.02%), 2-ethylhexanol (2.1%), phenylacetone (1.36%), and dimethyl trisulfide (1.04%). Therefore, dimethyl disulfide, cyclohexyl(dimethoxy)methylsilane and dimethyl trisulfide were selected for further analysis.

**Table 1:** Volatile organic compounds produced by LB and *Microbacterium maritypicum* Sneb159 detected by GC-MS analysis.

	Library/ID		CAS	RT	Area (%)	Qual
LB						
1	hexamethylcyclotrisiloxane		541-05-9	6.376	7.77	83
2	benzaldehyde		100-52-7	11.305	21.17	97
3	octamethylcyclotetrasiloxane		556-67-2	12.577	38.5	86
4	phenylacetaldehyde		122-78-1	14.46	0.24	93
5	2,5-dimethyl-3-ethylpyrazine		13360-65-1	15.733	0.22	86
6	decamethylcyclopentasiloxane		541-02-6	18.349	19.06	95
7	dodecamethylcyclohexasiloxane		540-97-6	24.333	10.44	91
8	tetradecamethylcycloheptasiloxane		107-50-6	29.794	3.13	93
9	2,4-di-tert-butylphenol		96-76-4	30.76	0.92	96
*M. maritypicum* Sneb159						
1		dimethyl disulfide	624-92-0	4.483	50.25	96
2		hexamethylcyclotrisiloxane	541-05-9	6.438	1.19	90
3		2,5-dimethyl pyrazine	123-32-0	9.455	0.49	86
4		dimethyl trisulfide	3658-80-8	11.472	1.04	95
5		2-ethylhexanol	104-76-7	13.933	2.1	83
6		3-ethyl-2,5-dimethylpyrazine	13360-65-1	15.727	0.09	81
7		phenylacetone	103-79-7	17.605	1.36	91
8		cyclohexyl(dimethoxy)methylsilane	17865-32-6	18.338	10.02	95
9		dodecamethylcyclohexasiloxane	540-97-6	24.333	3.82	91
10		tetradecamethylcycloheptasiloxane	107-50-6	29.788	1	93
11		2,4-di-tert-butylphenol	96-76-4	30.755	0.24	96

### Contact nematicidal effect of commercial compounds on nematode eggs and juveniles

**Figure 6:**
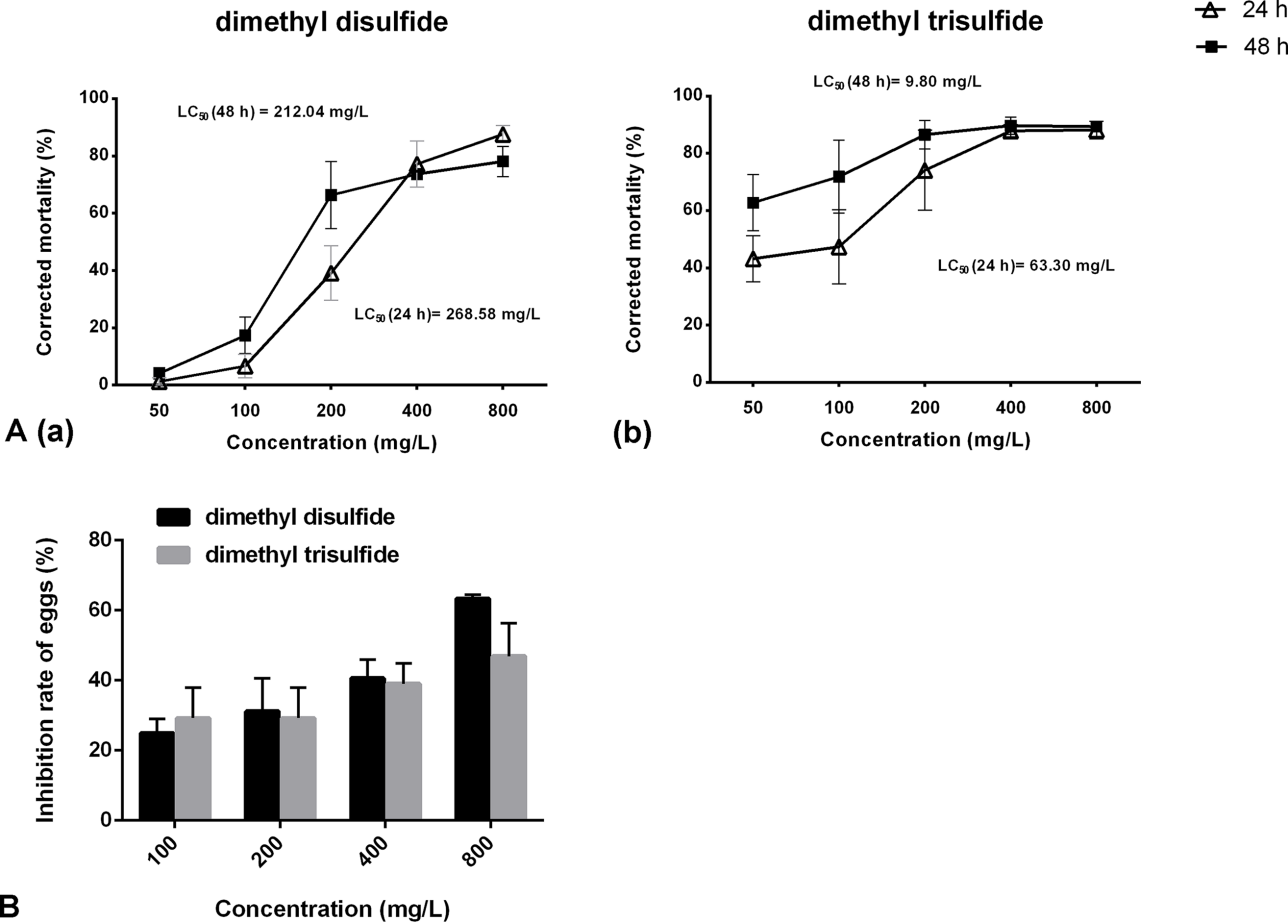
Contact effect of commercial compounds on *Heterodera glycines* juveniles and eggs. **(A)** Nematicidal activity of dimethyl disulfide (a) and dimethyl trisulfide (b) against *H. glycines*. **(B)** Inhibition effects of dimethyl disulfide and dimethyl trisulfide on the hatchability of *H*. *glycines* after 9 days.

The nematicidal activity of dimethyl disulfide and dimethyl trisulfide against *H. glycines* was determined using LC_50_/24 h values of 268.58 and 63.3 mg/L. The LC_50_ value for 48 h was 212.04 mg/L for dimethyl disulfide and 9.80 mg/L for dimethyl trisulfide (**Figure 6A**). No nematicidal activity was observed for cyclohexyl(dimethoxy)methylsilane with a mortality rate of less than 12% even at a concentration of 800 mg/L (data not shown).

*In vitro* exposure of *H. glycines* eggs to dimethyl disulfide and dimethyl trisulfide resulted in a reduction in the number of hatched J2s. The inhibition rate of dimethyl disulfide varied from 24.94% to 63.36% as the concentration ranged from 100 mg/L to 800 mg/L. A hatching reduction of 46.87% was observed at a concentration of 800 mg/L for dimethyl trisulfide (**Figure 6B**).

### Fumigation nematicidal activity of commercial volatiles to *H. glycines* eggs and juveniles

**Figure 7:**
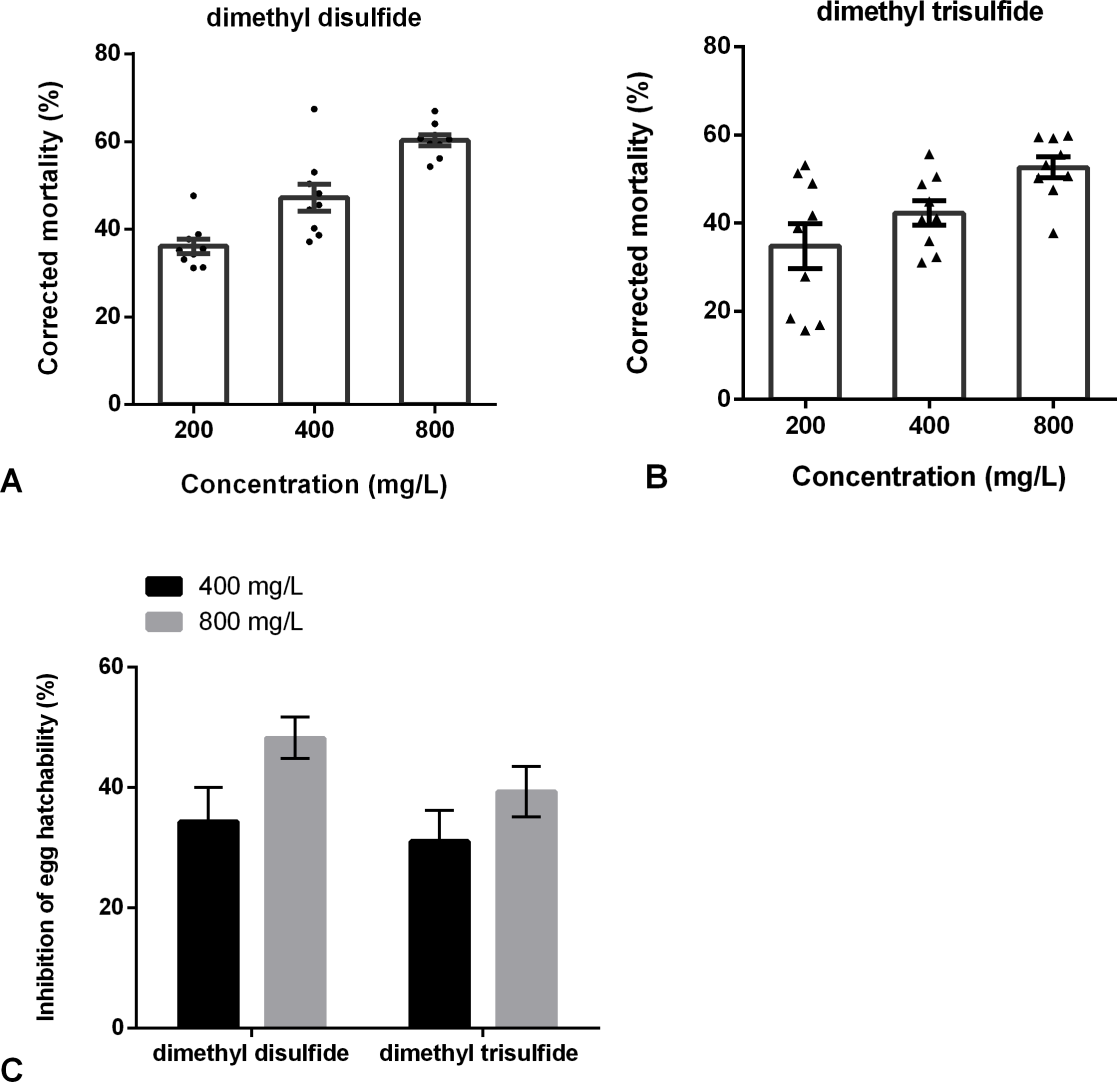
Fumigation effects of commercial VOCs on *Heterodera glycines*. **(A)** Fumigation effects of dimethyl disulfide on juveniles for 72 h. **(B)** Fumigation effects of dimethyl trisulfide on juveniles for 72 h. **(C)** Fumigation effects of dimethyl disulfide and dimethyl trisulfide on eggs hatching after 9 days. Data are expressed as the average of 9 replicates ± standard error.

The fumigant activities of dimethyl disulfide and dimethyl trisulfide against *H. glycines* were determined. The results showed that the corrected mortality of dimethyl disulfide at concentrations of 400 and 800 mg/L was 47.02% and 60.32% at 72 h, respectively. And the corrected mortality of dimethyl trisulfide was 52.63% at 800 mg/L after 72 h exposure (**Figure 7**). Besides, dimethyl disulfide and dimethyl trisulfide have a negative effect on egg hatching. The inhibition of dimethyl disulfide at 400 and 800 mg/L was 34.34% and 48.28%; and a 39.38% reduction in egg hatchability of dimethyl trisulfide at 800 mg/L (**Figure 7**).

### Genome feature of *M. maritypicum* Sneb159

**Figure 8:**
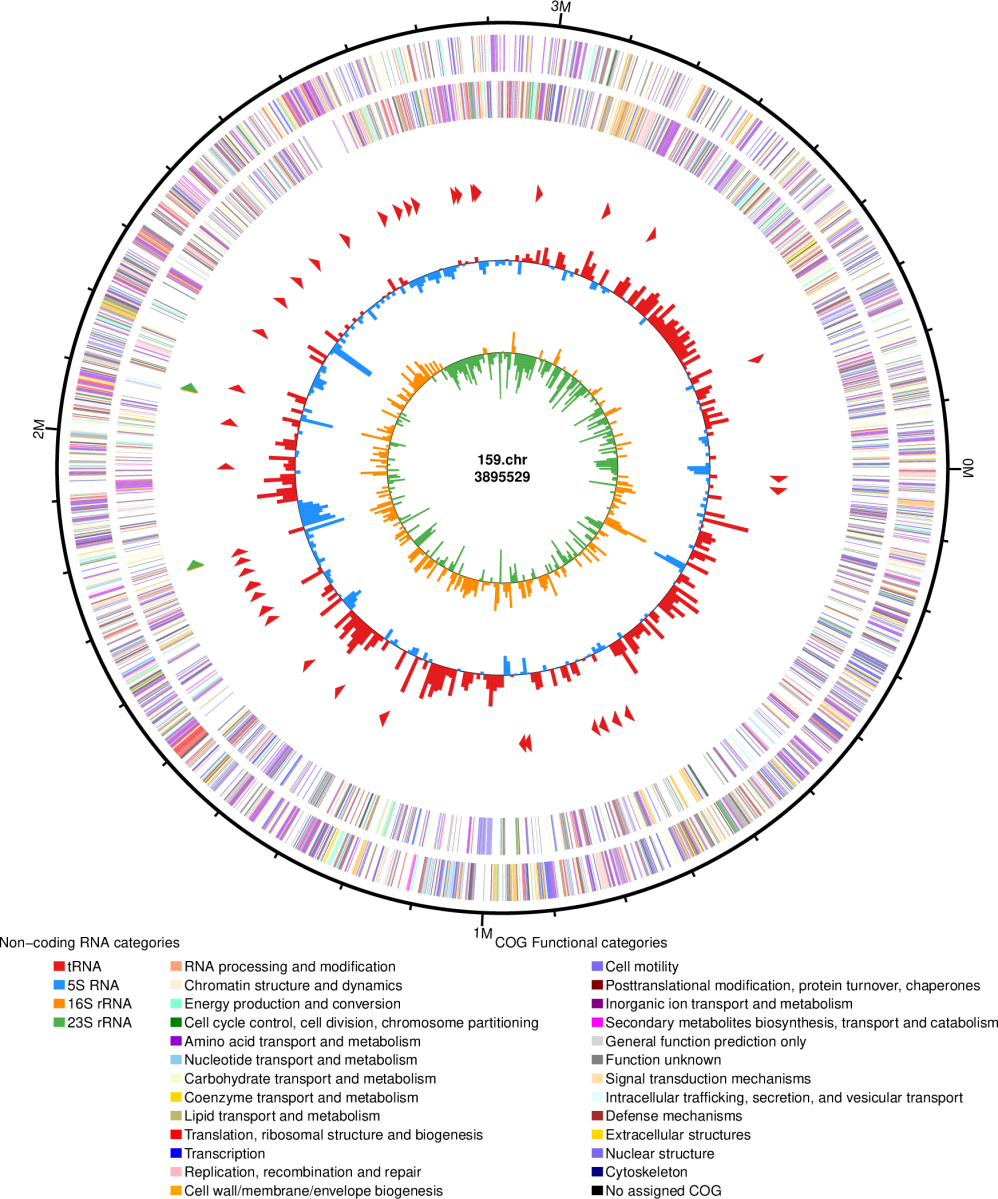
Overview of the *Microbacterium maritypicum* Sneb159 genome. From outside to inside: (1) The outer circle is the position coordinate of genome sequence. (2-3) Predicted coding sequences of the forward strands and reverse strands. (4) Positions of rRNA and tRNA. (5) Content of GC. (6) GC skew.

To better understand the genomic features of M. maritypicum Sneb159, the third generation was conducted by combining the Illumina TruSeq system and PacBio Sequel II high-throughput sequencing technologies. The genome assemblies of Sneb159 showed a 3.89 Mb genome with an average G + C content of 68.63%, consisting of one circular chromosome (**Table 2**, **Figure 8**). In total, 3,746 protein-coding genes with an average gene length of 965 bp were present in Sneb159. In addition, 6 rRNA genes and 48 tRNA genes were also identified. The genome sequence data have been uploaded to the GenBank database with accession number CP168030.

**Table 2:** General features and functional annotation of *Microbacterium maritypicum* Sneb159.

Genomic feature	Value
Genome size (bp)	3,895,529
G+C content (%)	68.63
rRNA genes	6
tRNA genes	48
Protein-coding genes	3746
Mean of gene length (bp)	965
Genes assigned to COG	3542
Virulence factors	7
Transport proteins	715
Antibiotic Resistance genes	75
Metal resistance genes	396

### Functional annotation of *M. maritypicum* Sneb159

**Figure 9:**
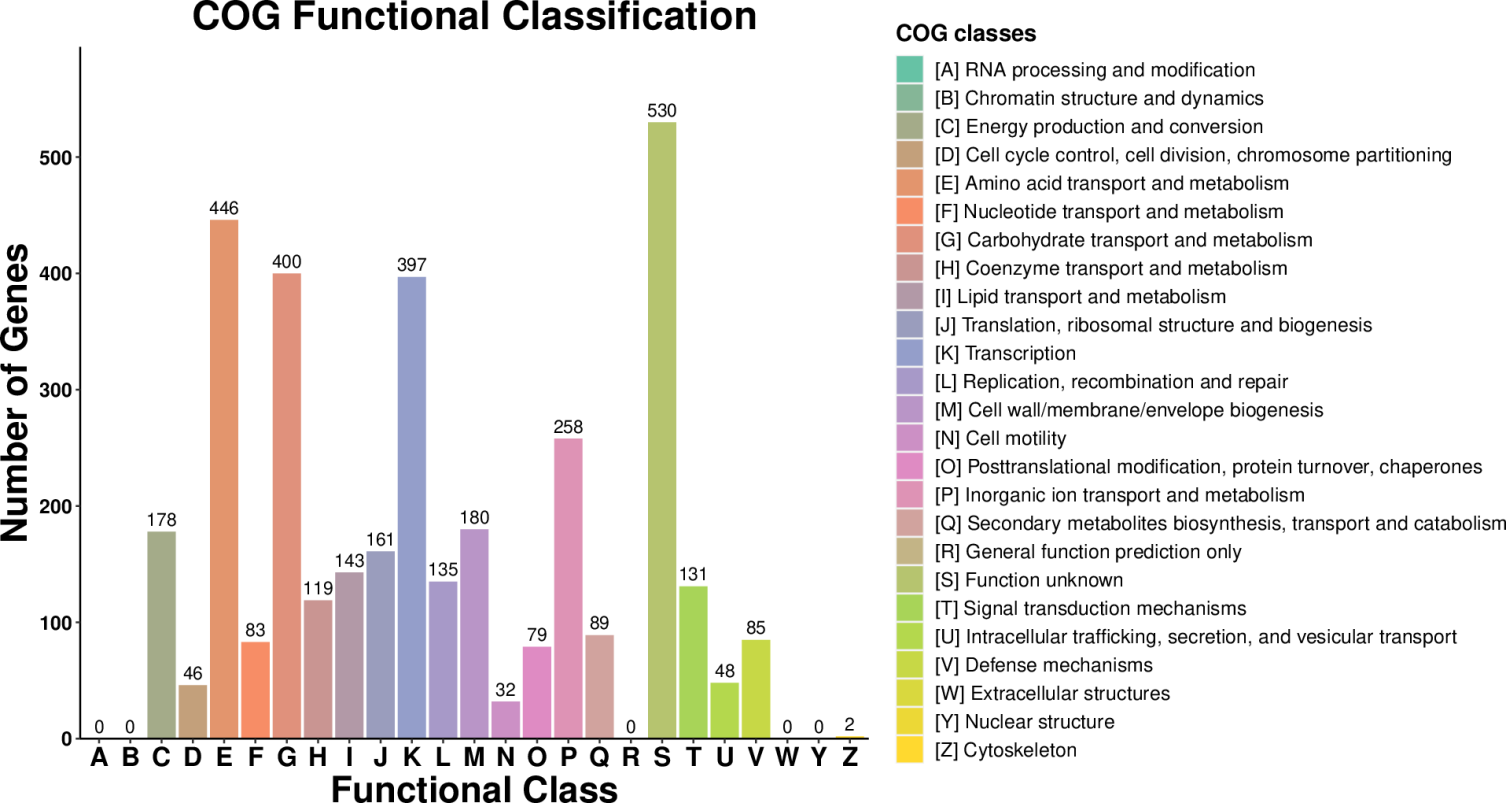
COG function analysis of *Microbacterium maritypicum* Sneb159.

A total of 3161 CDS were assigned to COG (Clusters of Orthologous Groups) by EggNOG. The functional classification of COG showed that the largest proportions were unknown functions, which accounted for 14.96% of the entire CDSs (**Figure 9**). Genes related to amino acid transport and metabolism were the second largest, with 12.59%, and 400 genes related to carbohydrate transport and metabolism accounted for 11.29%. 397 genes related to the transcript and 258 genes related to inorganic ion transport and metabolism were also commented on. It has been demonstrated that the transport and metabolism of amino acids and carbohydrates are fundamental to all life entities and play a crucial role in sustaining life activities.

Groups of genes that may be responsible for the virulence of the strain were investigated. The *M. maritypicum* Sneb159 genome was annotated with 715, 75, and 7 genes for known transporters, antibiotic resistance genes, and virulence factors. Most of them were associated with resistance to macrolides (55.7%), glycopeptides, and peptides (7.1%) (**Table 2**).

### Identification of secondary metabolite gene clusters

Three putative biosynthetic gene clusters of *M. maritypicum* Sneb159 were predicted in the genome using antiSMASH. They were assigned to three types, including hydrogen-cyanide, terpene, and T3PKS. In two established biosynthetic gene clusters, carotenoid and frankobactin, the similarity was 28% and 12%, respectively. In the results of comparison with known gene clusters, one gene cluster showed 50% similarity with the cluster carotenoid, the other one showed 12% homology with the cluster frankobactin, and one failed to match the known cluster.

## Discussion

*H. glycines* is a critical plant pathogen that continues to spread and causes severe damage in soybean-growing regions globally (Schmitt et al., 2004). Many chemicals are banned due to the side effects of environmental pollution, poisoning of humans and animals. Accordingly, more and more studies are being conducted to use natural products, which are produced by microorganisms and plants (Sun et al., 2022; Park et al., 2023). Bacteria are considered an important microbial resource, producing bioactive metabolites that palsy or even kill nematodes. The natural products fabclavines, rhabdopeptides, and xenocoumacins from *Xenorhabdus* bacteria, which form a symbiotic relationship with *Steinernema* nematodes, were highly toxic to both *C. elegans* and *M. javanica* (Abebew et al., 2022). The *Microbacterium* strain was isolated from the marine sample, further characterized by Takeuchi and Hatano, and transferred from *Flavobacterium marinotypicum* Zobell and Upham to the genus *Microbacterium maritypicum* comb. nov (Takeuchi and Hatano, 1998). *M. maritypicum* Sneb159 was isolated from the rhizosphere soil of soybeans (Zhao et al., 2019). In the present, the culture of *M. maritypicum* Sneb159 showed a high degree of nematicidal activity. No significant mortality was found in the treatment of bacterial cells, which suggested that the nematicidal active components are present in the supernatants. Jin et al. (2019) reported that the corrected J2s mortalities of *H*. *glycines* from the 2-fold dilution of culture filtrates of *Aspergillus niger* NBC001, *Penicillium oxalicum* NBC008 and *P. oxalicum* NBC012 were 88%, 90% and 92% respectively. However, the corrected mortality from the 10-fold dilution of Sneb159 was 87.25%.

In the pot experiment, culture filtrate exhibited a significant decrease in *H. glycines* infection and development compared to the control, and the antagonistic activity decreased as dilution increased. Martinuz et al. (2013) evaluated the *in vivo* effect of *Fusarium oxysporum* Fo162 on reduced nematode penetration and delayed development. *Streptomyces plicatus* G produced volatile compounds that influence the chemotactic behavior of *M. incognita* (Wang et al., 2019). Seed coated with *B. megaterium* Sneb207, *Klebsiella pneumoniae* SnebYK, and *Sinorhizobium fredii* Sneb183 decreased the penetration of *H. glycines* with reduction rates of 39.02%, 47.32%, and 40.13%, respectively (Tian et al., 2014; Liu et al., 2018; Zhou et al., 2021). In our study, the reduction of Sneb159 culture filtrate was 70.67%, and still reached 56.88% when diluted 100-fold by using the root irrigation treatment.

In the present study, Sneb159 exhibited repellent activities towards *H. glycines*, and more nematodes were driven in the opposite direction of the Sneb159 culture treatment. No significant difference between the total number of nematodes in the Sneb159 treatment and control, which inferred that the reduction in nematode numbers on the Sneb159 was due to repelling rather than killing. The volatile metabolites produced by certain bacteria were found to be associated with the chemotaxis of *M. incognita*. The VOC decanal was observed to regulate the movement of *M. incognita* in pots with or without plants (Li et al., 2023). It’s also conceivable that Sneb159 exerted an impact on the root exudate metabolites of soybean to participate in the nematode infection, the relationship between soybean, Sneb159, and *H. glycines* needs further exploration.

Bacteria (particularly *Pseudomonas* and *Bacillus*) and plants belonging to families such as Asteraceae, Myrtaceae, Brassicaceae, and Rutaceae are known to produce nematicidal VOCs (Deng et al., 2022). The nematicidal activity of *B. cereus* Bc-cm103 VOCs was detected, and 21 compounds have been identified (Yin et al., 2021). In this study, the VOCs of *M. maritypicum* Sneb159 exert a strong inhibitory effect on the activity of *H. glycines* J2s and egg hatching. Chen et al. (2022) reported that the VOCs of *B. aryabhattai* MCCC 1K02966 showed contact nematicidal, fumigant, as well as repellent activity against *M. incognita*. Here, 7 VOCs were identified from *M. maritypicum* Sneb159. To the best of our knowledge, no reports exist concerning the VOC metabolite profile of *M. maritypicum*. Dimethyl disulfide and dimethyl trisulfide showed a contract and fumigate effect on juveniles and eggs. Agisha et al. (2019) found that dimethyl trisulfide inhibited *Pythium myriotylum*, *Phytophthora capsica*, and *Rhizoctonia solani* when the concentration was 6.25 mg/L, and had nematicidal activity against *Radopholus similis* at 2 g/L, which was more effective than carbosulfan. Furthermore, *Pseudoalteromonas marina* H-42 and *Vibrio atlanticus* S-16 were identified to produce dimethyl trisulfide, which exhibited an LC_90_ value of 0.060 mmol/L against *B. xylophilus* (Yu et al., 2015). Similarly, the 48 h LC_50_ value for dimethyl trisulfide against *H. glycines* was 9.80 mg/L. Tang et al. (2024) found that the expression of ergosterol biosynthesis-related genes *Cgerg6* and *Cgerg11* of *Colletotrichum gloeosporioides* was significantly suppressed when they were exposed to dimethyl trisulfide, which are involved in an interaction with the antifungal activity of dimethyl trisulfide. However, few studies were focused on the nematicidal mechanism of dimethyl trisulfide.

Dimethyl disulfide is a volatile substance widely produced in Alliaceae, Brassicaceae, soil bacteria, and fungi (Giorgio et al., 2015; Kassam et al., 2021). Considered an alternative to methyl bromide, dimethyl disulfide is a volatile compound without ozone depletion potential (Gillis, 2003; Fritsch, 2005). It was observed that fumigation with dimethyl disulfide resulted in the mortality of *M. incognita* juveniles and demonstrated an effectiveness of 80–94% in field experiments on tomatoes at concentrations ranging from 10 to 100 g/m^2^ (Yan et al., 2019). Furthermore, dimethyl disulfide promoted the growth of tomatoes and caused significant increases in root and shoot length, fresh and dry weight, leaf area, and chlorophyll content. The upregulation of *PR1* and *PR5* after dimethyl disulfide treatment indicated that the systemic resistance mediated by the SA signaling pathway was induced in tomatoes (Tyagi et al., 2020). Dimethyl disulfide expedited the oxygen consumption of nematode while inhibiting acetylcholinesterase activity. The mechanisms of action for contact and fumigation treatments with DMDS differ significantly. Contact exposure to DMDS triggers a transient influx of calcium ions, which affects nematode muscle contractions and leads to the disintegration of body wall structures. On the other hand, fumigation with DMDS effectively modulates the release of nematode neurotransmitters, thereby influencing the nervous system of the nematodes (Wang et al., 2023).

According to previous studies, the mechanisms of biocontrol agents antagonism against *H. glycines* induced systemic resistance by triggering SA and JA pathways, and directly killed by secondary metabolites (Liu et al., 2018; Zhou et al., 2021). Our previous study has reported that seed-coated treatment induced the local and systemic resistance of soybeans (Zhao et al., 2019). Above all, Sneb159 is a novel and multifunctional agent, the remarkable control efficiency on *H. glycines* is a comprehensive action of repellency, killing by metabolites and VOCs, as well as induction of resistance. This means it’s an excellent biocontrol resource for plant parasitic nematodes. Therefore, the application of seed coated with a minimal amount of fermentation broth to induce soybean resistance, followed by root irrigation to expel and eliminate the nematode population in the soil, may yield enhanced efficacy in field applications. This approach requires further confirmation in future studies. Additionally, unresolved issues include the large-scale fermentation of the Sneb159, extending the shelf life of the active fermentation broth, evaluating the impact on the rhizosphere soil microbial communities, and assessing the safety to the environment and mammals.

The genome of *M. maritypicum* Sneb159 was predicted to contain three secondary metabolite synthesis gene clusters by antiSMASH software, including hydrogen-cyanide, terpene, and T3PKS. Lenchi (2023) found that carotenoid was the most similar cluster for *Microbacterium* strains *M. oxydans* and *M. maritypicum*. Carotenoids are a diverse group of lipid-soluble pigments, which is the reason for the yellow colony of *M. maritypicum* Sneb159. Besides, carotenoids are involved in the membrane fluidity and protection of cells from oxidative damage and UV radiation (Mohana et al., 2013). Carotenoid from marine *B. infantis* presented excellent antimicrobial and antioxidant activity, which was against *Pseudomonas aeruginosa*, *Shigella dysenteriae*, *Salmonella enterica ser. typhi*, *Serratia marcescens*, *B. megaterium*, *Staphylococcus aureus*, and *S. epidermis* (Soni et al., 2023). Carotenoids are tetra-terpenoids that exert a direct effect on the cell wall and membrane of bacteria, leading to membrane disruption and leakage of cellular contents (Rizk et al., 2021). Although antibacterial activity has been discovered, the relationship between carotenoid and nematicidal activity remains unclear. Frankobactin A1 has the ability to detoxify Cu (II), preventing its cellular entry. It plays an important role in the symbiosis of the host and the reclamation of copper-contaminated soil (Mohr et al., 2021). Although the secondary metabolite synthesis gene clusters are not sufficient to discern the nematicidal activity, it suggests that Sneb159 has the potential to produce some new metabolites with novel biological functions, and the nematicidal traits need further study.

In summary, the fermentation filtrate of *M. maritypicum* Sneb159 rather than bacterial cells has nematicidal characteristics against *H. glycines*. The fermentation filtrate suppressed the infection and development of *H. glycines*. Furthermore, the repellent effect of *M. maritypicum* Sneb159 was observed to reduce the infection number directly. Further analysis revealed that the VOCs produced by Sneb159 effectively reduced nematode activity and inhibited egg hatching, and that dimethyl disulfide and dimethyl trisulfide were active in both the soaking and fumigation treatments. So far, it is the first complete genome sequence of a nematicidal *M. maritypicum*, although draft genomes of *M. maritypicum* with other abilities have been submitted to NCBI. Further research is required to elucidate the relationship between metabolites, gene clusters, and nematicidal activity.

## Data Availability

The datasets presented in this study can be found in online repositories. The names of the repository/repositories and accession number(s) can be found below: https://www.ncbi.nlm.nih.gov/genbank/, CP168030.
